# Effects of Caffeine–Taurine Co-Supplementation on Exercise Performance: A Systematic Review and Meta-Analysis

**DOI:** 10.3390/nu18203434

**Published:** 2026-10-20

**Authors:** Bingbing He, Chengyu Zhou, Zhuo Zeng, Xusong Dong, Dongxu Huang, Haoqiang Shi, Haoran Li, Kai Gao, Yongmin Xie, Aiguo Zhou, Diandong Lang, Qinshuo Zhang

**Affiliations:** 1 School of Physical Education and Sports, Beijing Normal University, Beijing 100875, China; 2022420077@bnu.edu.cn; 2 School of Strength and Conditioning Training, Beijing Sport University, Beijing 100084, China; 2021240897@bsu.edu.cn (C.Z.); 2024210137@bsu.edu.cn (Z.Z.); 2023012202@bsu.edu.cn (X.D.); dongxuhuang1027@163.com (D.H.); m15966507473@163.com (H.S.); marslee_24@163.com (H.L.); xieyongminbsu@163.com (Y.X.); aiguozhou@126.com (A.Z.); 3 College of Sports and Health, Linyi University, Linyi 276005, China; tylp7658@163.com (K.G.); langdiandong123@126.com (D.L.); 4 Department of Sports Science, The University of Suwon, Hwaseong-si 18323, Republic of Korea

**Keywords:** taurine, exercise performance, caffeine, systematic review, meta-analysis

## Abstract

**Background:** Caffeine–taurine combinations are widely used in commercial sports products. Whether this combination improves exercise performance is still unclear. This study aimed to estimate the effect of caffeine–taurine co-supplementation versus placebo on exercise performance. **Methods:** Randomized controlled trials comparing caffeine plus taurine versus placebo were searched in PubMed, Embase, Web of Science, Cochrane Library, and related databases up to June 1, 2026. Outcomes included heart rate (HR), reaction time (RT), time to exhaustion (TTE), rating of perceived exertion (RPE), and peak/maximal anaerobic power (PP). A random-effects model was applied. Risk of bias was assessed with RoB 2.0. **Results:** A total of 11 randomized controlled trials involving 234 participants were included in the meta-analysis. Compared with placebo, caffeine–taurine co-supplementation was associated with improvements in time to exhaustion (SMD = 0.54, 95% CI: 0.03 to 1.05) and PP (SMD = 0.55, 95% CI: 0.21 to 0.89); an expanded secondary product-level analysis that additionally included one commercial multi-ingredient energy drink yielded a smaller estimate (SMD = 0.48, 95% CI: 0.19 to 0.78) that cannot be attributed specifically to caffeine and taurine. The pooled estimate for reaction time favored supplementation (SMD = −1.49, 95% CI: −2.78 to −0.20), but very high heterogeneity and a prediction interval crossing the null indicated that the effect was not consistent across settings, whereas no meaningful effect was observed for heart rate (WMD = −0.11, 95% CI: −5.76 to 5.53). Rating of perceived exertion was significantly reduced (SMD = −0.44, 95% CI: −0.83 to −0.04). **Conclusions:** Caffeine–taurine-only formulations were associated with possible benefits for selected performance outcomes, including PP. However, the evidence was derived from few, generally small studies and did not establish the independent contribution of caffeine, taurine, or their interaction. Results from commercial multi-ingredient products should be interpreted at the product level rather than as evidence of an isolated caffeine–taurine effect.

## 1. Introduction

Caffeine is one of the most used stimulants in sport and exercise contexts [1]. It is frequently taken before training or competition because of its perceived ability to improve alertness and physical performance [2]. Experimental and applied studies have generally reported beneficial effects on endurance capacity, perceived exertion, reaction time, and power output, although the magnitude of these effects is not entirely consistent across populations and protocols [3,4].

Taurine is also widely present in sports supplements and energy drinks. It participates in several physiological processes, including calcium handling, membrane stability, and oxidative regulation [5]. Some studies [6,7] suggest that taurine may influence muscle function and fatigue development, but the overall evidence is less robust compared with caffeine, and findings remain variable.

In commercial practice, caffeine and taurine are often consumed together. This combination is typically presented as having enhanced or complementary effects on physical and cognitive performance [8]. However, this assumption is not strongly supported by consistent experimental evidence. Individual trials have produced mixed results, with some reporting small performance benefits, while others show little or no difference compared with placebo. The inconsistency is evident across outcomes such as heart rate response, reaction time, time to exhaustion, perceived exertion, and power-related measures. Part of this inconsistency may be related to differences in study design, participant training status, exercise modality, and dosing strategies [9]. In addition, most evidence has been generated from small randomized trials, which limits the stability of conclusions when viewed in isolation [10].

Deng et al. [11]. conducted a Bayesian network meta-analysis comparing caffeine, taurine, and their combination, with a literature search through 25 July 2025. However, that study did not specifically focus on direct comparisons of caffeine–taurine co-supplementation versus placebo for individual exercise outcomes. Therefore, we updated the search to 1 June 2026 and performed a pairwise meta-analysis of time to exhaustion, peak/maximal anaerobic power, reaction time, heart rate, and perceived exertion. This analysis complements the previous network meta-analysis but cannot determine the independent or synergistic effects of caffeine and taurine. Given these uncertainties, a pooled quantitative assessment focusing specifically on caffeine–taurine co-supplementation versus placebo is needed. This study therefore aimed to synthesize randomized controlled trials comparing the combined intervention with placebo and to estimate differences in heart rate, reaction time, time to exhaustion, rating of perceived exertion, and PP. Because the planned contrasts did not compare co-supplementation with caffeine alone or taurine alone, this meta-analysis was not designed to determine the independent contribution of either ingredient or a caffeine–taurine interaction.

## 2. Materials and Methods

### 2.1. Search Strategy and Study Selection

This systematic review and meta-analysis were conducted in accordance with PRISMA guidelines [12]. The completed PRISMA 2020 checklist is provided as Supplementary File S1. The review was first submitted to PROSPERO on 25 June 2026 (CRD420261432477). The literature search had been conducted before registration; however, title and abstract screening, full-text assessment, data extraction, and statistical analysis had not commenced at the time of registration.

PubMed, Embase, Web of Science Core Collection, and the Cochrane Central Register of Controlled Trials (CENTRAL) via the Cochrane Library were searched from database inception to 1 June 2026. The search combined controlled-vocabulary and free-text terms related to caffeine, taurine, energy drinks, exercise, physical and cognitive performance, and randomized trials. No language restrictions were applied, and no additional bibliographic databases were searched. The complete database-specific search strategies, including all search terms, Boolean operators, field codes, limits, and platforms, are provided in Supplementary Table S1.

The search strategy combined terms related to caffeine, taurine, and exercise performance, together with randomized controlled trial filters. Reference lists of retrieved articles and relevant reviews were also screened manually to identify additional eligible studies. Two reviewers independently screened titles and abstracts, followed by full-text assessment. Disagreements were resolved through discussion, and when necessary, by consultation with a third reviewer.

### 2.2. Eligibility Criteria

Studies were included:

(1) Randomized controlled trial design.
(2) Compared a formulation containing both caffeine and taurine with a concurrent placebo or control condition. Eligible interventions could contain caffeine and taurine alone or as components of a multi-ingredient formulation. Effects from multi-ingredient formulations were interpreted at the product-formulation level and were not attributed exclusively to caffeine and taurine.
(3) Involved healthy, trained, or physically active participants.
(4) Reported at least one of the predefined outcomes.
(5) For multi-arm studies, only groups relevant to the prespecified comparison were extracted. Caffeine-only and taurine-only groups were excluded. For the primary quantitative synthesis, we included only outcome-specific comparisons in which caffeine and taurine were the only potentially active ingredients differentially administered between the intervention and comparator. Balanced factorial co-interventions were permitted when the co-intervention was applied equally across the relevant caffeine–taurine and control conditions. Commercial multi-ingredient formulations containing additional unmatched potentially active constituents were excluded from the primary analysis and evaluated separately in expanded secondary product-level analyses.

Studies were excluded if they were non-randomized, animal experiments, lacked a placebo control, or did not provide sufficient data for effect size calculation.

Outcomes: The primary outcomes included time to exhaustion (TTE) and PP (PP). Secondary outcomes were heart rate (HR), reaction time (RT), and rating of perceived exertion (RPE).

### 2.3. Data Extraction and Quality Assessment

Data were independently extracted by two investigators using a standardized form. Extracted variables included study characteristics, participant information, intervention details, intervention delivery format (capsule, laboratory-prepared beverage, or commercial energy drink) and outcome.

Risk of bias was assessed at the result level. The RoB 2 tool for randomized crossover trials (18 March 2021 version) [13] was applied to the ten crossover trials, including Domain S for bias arising from period and carryover effects. Treatment-sequence allocation and balance, counterbalancing, washout or visit spacing, pretrial restrictions, missing periods, within-participant analysis, and any reported order, period, or carryover assessment were considered. Mizera et al., the parallel-group trial, was assessed using the standard RoB 2 tool for parallel randomized trials. Two reviewers conducted the assessments independently, with disagreements resolved through discussion. The certainty of evidence for each outcome was assessed using the GRADE approach, considering risk of bias, inconsistency, indirectness, imprecision, and publication bias. Randomized evidence initially received a high-certainty rating and was downgraded when important limitations were identified.

### 2.4. Statistical Analysis

Meta-analysis was conducted using a random-effects model due to expected clinical and methodological variability across studies. For crossover trials, the paired nature of the data was preserved. When the standard deviation of within-participant differences was unavailable, it was calculated as:

SD_diff = √(SD_I^2^ + SD_C^2^ − 2r × SD_I × SD_C).

Correlation coefficients were derived from reported paired statistics where possible; otherwise, r = 0.50 was imputed. Sensitivity analyses were conducted using r = 0.25 and 0.75. Effect estimates and corresponding standard errors were pooled using a random-effects generic inverse-variance model.

Heterogeneity was assessed using the *I*^2^ statistic and interpreted according to Cochrane guidance: 0–40% might not be important, 30–60% may represent moderate heterogeneity, 50–90% may represent substantial heterogeneity, and 75–100% may represent considerable heterogeneity. Because these ranges overlap, *I*^2^ values were also interpreted in relation to the magnitude and direction of effects and the strength of evidence for heterogeneity. When substantial or considerable heterogeneity was detected, the results were interpreted with caution.

HR was summarized using the weighted mean difference (WMD). RT, TTE, PP, and RPE were summarized using Hedges’ g, that is, the standardized mean difference with the conventional small-sample bias correction factor (J), because these outcomes were reported using different numerical scales across studies. Positive SMD values favored supplementation for TTE and PP, whereas negative SMD values favored supplementation for RT and RPE.

Subgroup analyses were performed to explore potential sources of heterogeneity. Analyses were stratified according to sex distribution, participant characteristics, and supplementation dose. All subgroup and meta-regression analyses were considered exploratory and hypothesis-generating. Results from sparse or single-study strata were treated as descriptive, and non-significant interaction tests were not interpreted as evidence of no effect modification.

Leave-one-out sensitivity analyses were conducted by sequentially omitting each study. Subgroup and meta-regression analyses were considered exploratory and hypothesis-generating. Funnel plots and Egger’s regression tests were planned only for outcomes including at least 10 studies. Because no outcome met this threshold, these analyses were not performed. All analyses were performed using Stata/MP version 19.0, with two-sided *p* < 0.05 indicating statistical significance.

## 3. Results

### 3.1. Study Selection

**Figure 1:**
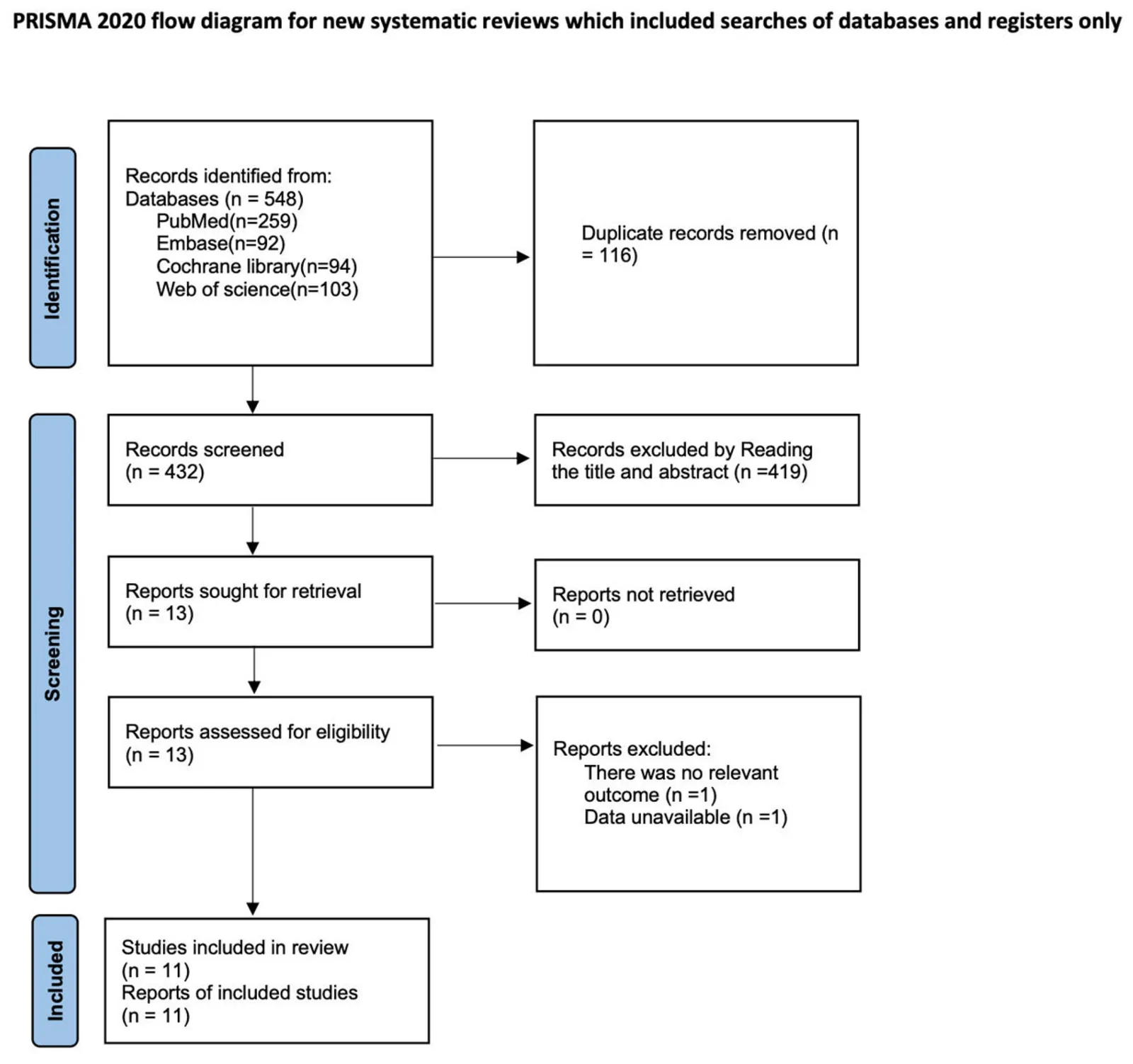
PRISMA flow diagram.

The initial search identified a total of 548 records from four databases, including PubMed (*n* = 259), Embase (*n* = 92), Cochrane Library (*n* = 94), and Web of Science (*n* = 103). After removing 116 duplicate records, 419 records were excluded due to irrelevance to the study question based on titles and abstracts. 2 studies were excluded by reading full text (The full citations and specific reasons for exclusion are presented in Supplementary Table S2). Ultimately, 11 randomized controlled trials [14,15,16,17,18,19,20,21,22,23,24] were included in meta-analysis (Figure 1).

### 3.2. Study Characteristics

A total of 11 randomized controlled trials involved 234 participants. The overall population consisted mainly of young healthy adults, with mean ages ranging from approximately 19.7 to 24 years across studies. Regarding sex distribution, most studies enrolled male participants or male-dominant samples. Two studies included female-only cohorts, while a smaller number reported mixed-sex populations. Nine studies administered caffeine and taurine as isolated ingredients in capsule-based preparations without additional active ingredients in the analyzed intervention arm. Giles et al. administered caffeine and taurine in capsules together with either a glucose-containing or matched non-glucose beverage in a factorial design. Gwacham et al. used AdvoCare Spark, a commercial multi-ingredient powdered energy drink containing caffeine, taurine, carbohydrate, vitamins, minerals, amino acids, and other potentially active constituents. In Mizera et al., only the taurine-plus-caffeine arm was included in the analyzed comparison; the separate phosphatidylserine-containing arm was excluded (Table 1). Detailed information on washout periods, administration timing, habitual caffeine consumption, pretrial restrictions, and exercise/testing protocols is presented in Supplementary Table S3.

### 3.3. Risk of Bias Assessment

**Figure 2:**
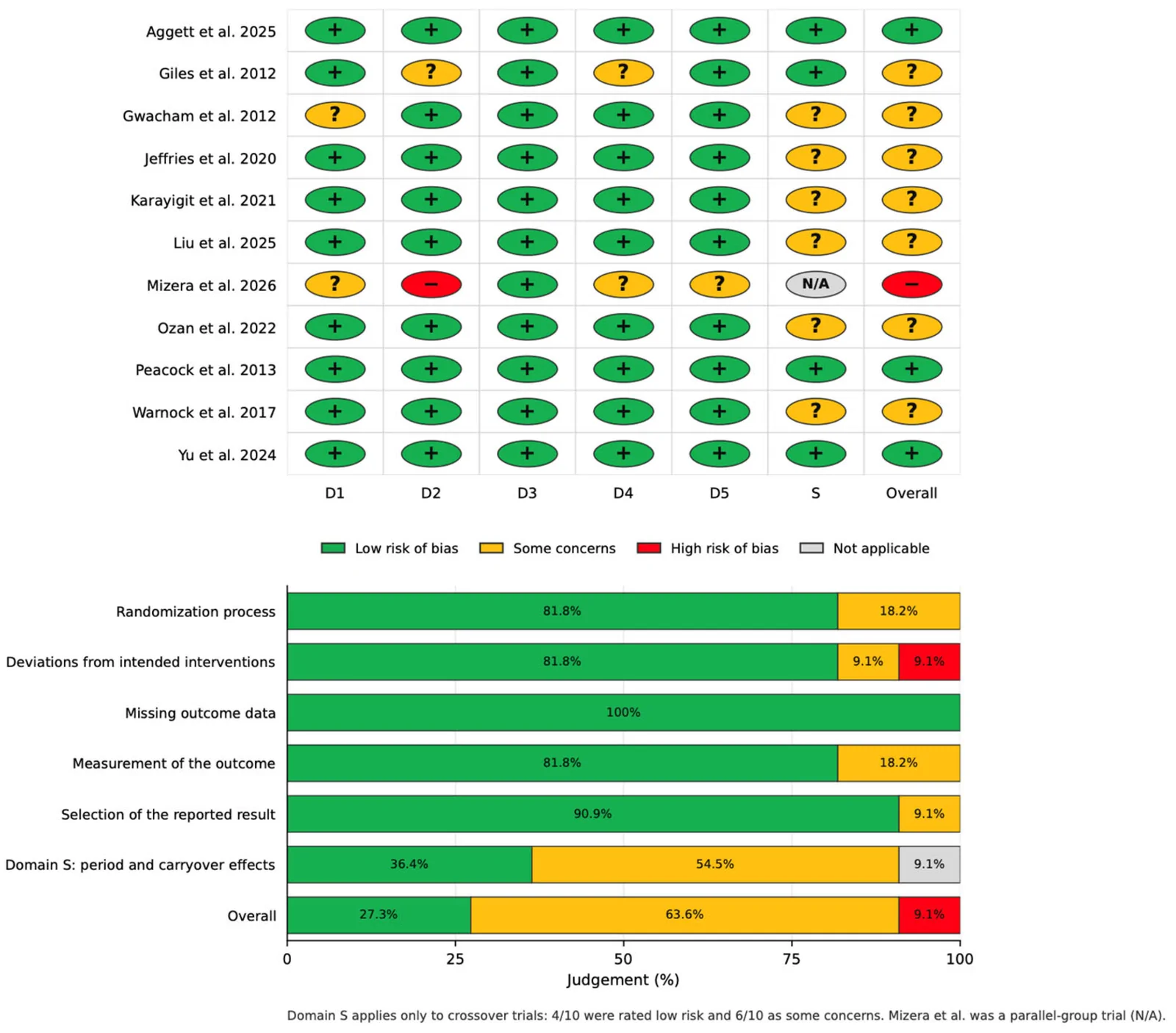
Risk-of-bias assessment of the included studies: Aggett et al. (2025) [14], Giles et al. (2012) [15], Gwacham et al. (2012) [16], Jeffries et al. (2020) [17], Karayigit et al. (2021) [18], Liu et al. (2025) [19], Mizera et al. (2026) [20], Ozan et al. (2022) [21], Peacock et al. (2013) [22], Warnock et al. (2017) [23], and Yu et al. (2024) [24]. Green indicates low risk of bias, yellow indicates some concerns, red indicates high risk of bias, and gray indicates that the domain was not applicable. Domain S evaluates potential period and carryover effects and applies only to crossover trials.

Ten studies adopted crossover designs. Eight reported a minimum washout interval of at least 48 h between experimental conditions. Liu et al. [19] stated that study visits were separated by no more than 3 days but did not specify a minimum interval. Ozan et al. [21] conducted four sessions within a 72-h period without explicitly reporting a washout interval. Balanced or counterbalanced treatment allocation was clearly described by Aggett et al. [14], Giles et al. [15], Peacock et al. [22], and Yu et al. [24]. Warnock et al. [23] found no evidence of an order effect on performance; however, none of the crossover studies reported a formal treatment-by-period interaction or carryover analysis. Consequently, inadequate reporting of sequence balance or washout procedures led to some concerns in the crossover-specific domain for several studies. Mizera et al. [20] was judged to be at high risk of bias arising from deviations from the intended interventions. The single-blind design did not ensure that investigators were unaware of group allocation, and knowledge of the assigned intervention could have influenced its delivery or the assessment of subjective outcomes. The overall risk-of-bias judgment for this study was therefore high. All included studies were considered to have a low risk of bias from missing outcome data. Figure 2 summarizes the risk-of-bias assessments, while domain-level judgments and their supporting reasons are reported in Supplementary Table S7. According to the GRADE assessment, the certainty of evidence was moderate for heart rate and rating of perceived exertion, low for time to exhaustion and peak power, and very low for reaction time (Supplementary Table S4).

### 3.4. Meta-Analysis Results

#### 3.4.1. Time to Exhaustion (TTE)

**Figure 3:**
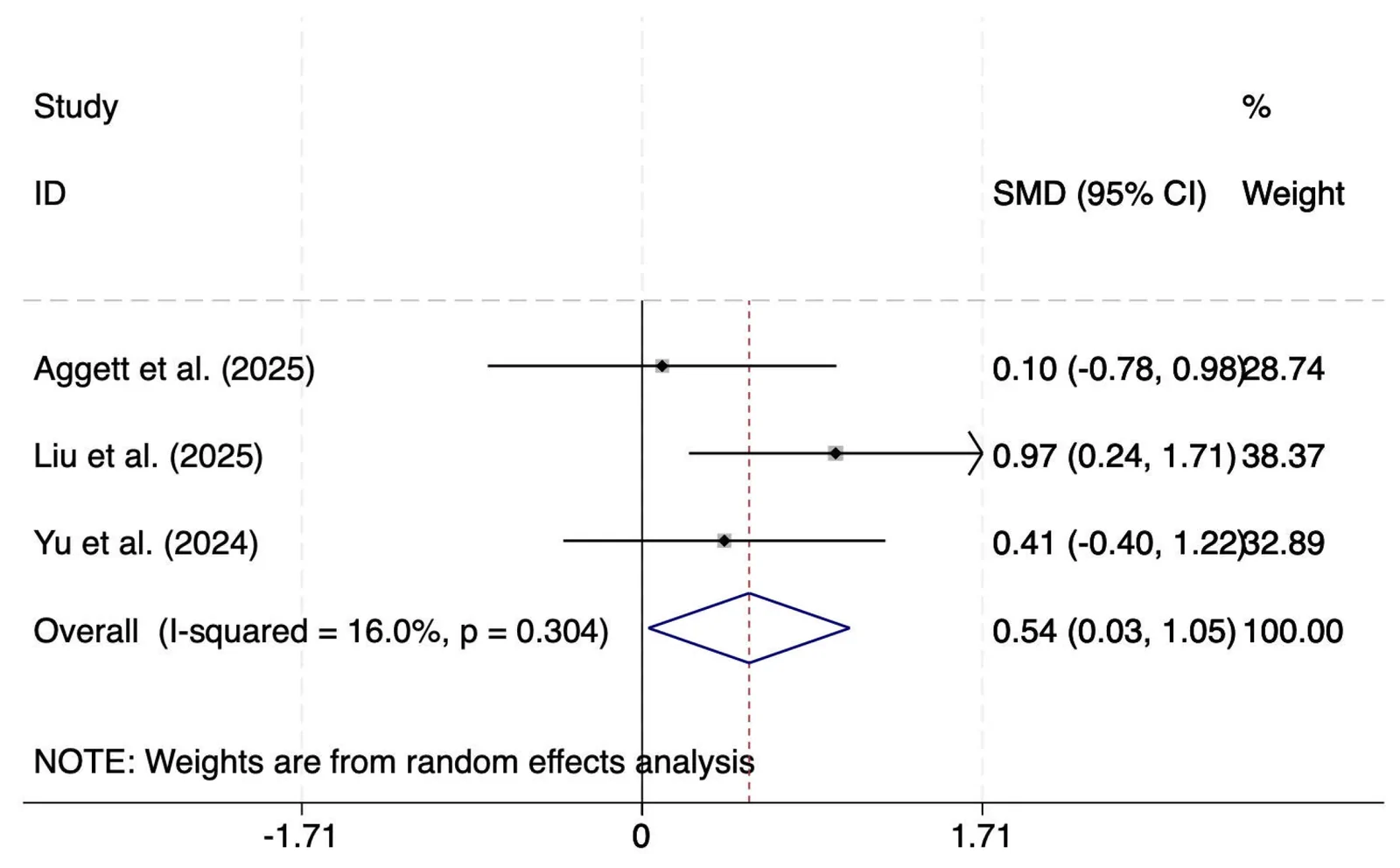
Effect of caffeine and taurine co-ingestion on time to exhaustion. Data were obtained from Aggett et al. (2025) [14], Liu et al. (2025) [19], and Yu et al. (2024) [24]. The solid vertical line at SMD = 0 represents the line of no effect, whereas the red dotted vertical line represents the pooled effect estimate from the random-effects model. Squares indicate study-specific effect estimates, with their sizes proportional to study weights; horizontal lines represent 95% CIs. The diamond represents the pooled SMD and its 95% CI. An arrow indicates that the corresponding confidence interval extends beyond the displayed plotting range. CI, confidence interval; SMD, standardized mean difference; TTE, time to exhaustion.

As shown in Figure 3, caffeine–taurine co-supplementation was associated with a significant improvement in TTE compared with placebo (SMD = 0.54, 95% CI: 0.03 to 1.05). Between-study heterogeneity was low (*I*^2^ = 16.0%), and individual study effects were generally consistent in direction.

#### 3.4.2. Peak/Maximal Anaerobic Power (PP)

To ensure construct consistency, the outcome was refined as “PP.” The exact variable, protocol, unit, and measurement time point extracted from each study are presented in Supplementary Table S8.

**Figure 4:**
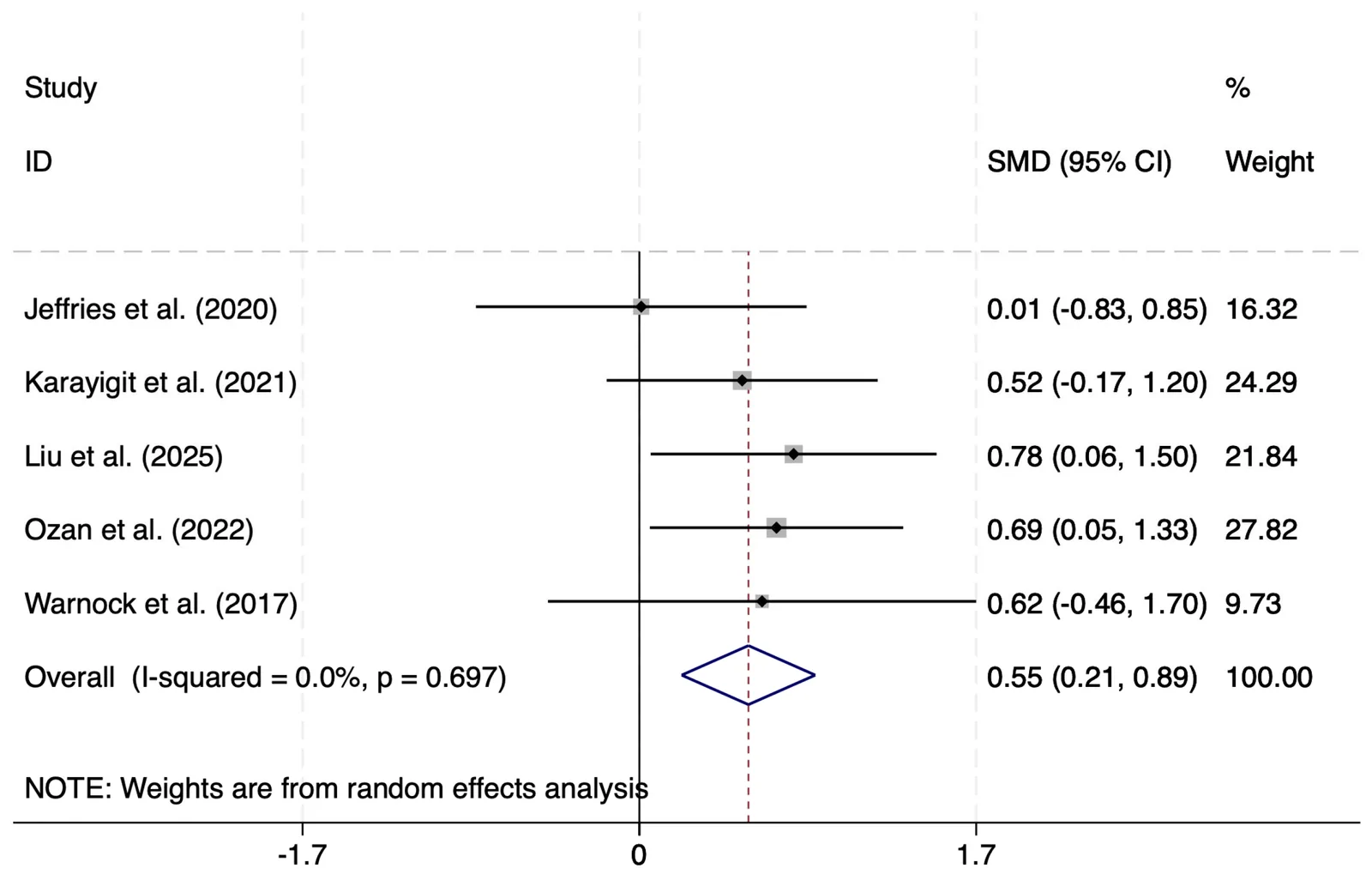
Primary meta-analysis of peak/maximal anaerobic power restricted to caffeine–taurine-only formulations. Data were obtained from Jeffries et al. (2020) [17], Karayigit et al. (2021) [18], Liu et al. (2025) [19], Ozan et al. (2022) [21], and Warnock et al. (2017) [23]. The solid vertical line at SMD = 0 represents the line of no effect, whereas the red dotted vertical line represents the pooled effect estimate from the random-effects model. Positive SMD values favor caffeine–taurine co-ingestion. Squares indicate study-specific estimates, with their sizes proportional to study weights; horizontal lines represent 95% CIs. The diamond represents the pooled SMD and its 95% CI. CI, confidence interval; SMD, standardized mean difference.

Figure 4 presents the pooled results for PP. A significant positive effect was observed following caffeine–taurine supplementation (SMD = 0.55, 95% CI: 0.21 to 0.89), with no detectable heterogeneity (*I*^2^ = 0%). Although effect sizes differed across trials, all studies tended to favor the intervention. Gwacham et al. was excluded from the primary analysis because the intervention was AdvoCare Spark, a commercial energy drink containing caffeine and taurine together with several additional constituents not fully matched in the comparator.

In an expanded secondary product-level analysis, Gwacham et al. was added to the five caffeine–taurine-only studies. The resulting pooled estimate was SMD = 0.48 (95% CI: 0.19 to 0.78; *I*^2^ = 0%; Supplementary Figure S2). Although the direction of association remained favorable, this expanded estimate represents the effect of caffeine–taurine-containing products and cannot be attributed specifically to caffeine and taurine.

#### 3.4.3. Heart Rate (HR)

**Figure 5:**
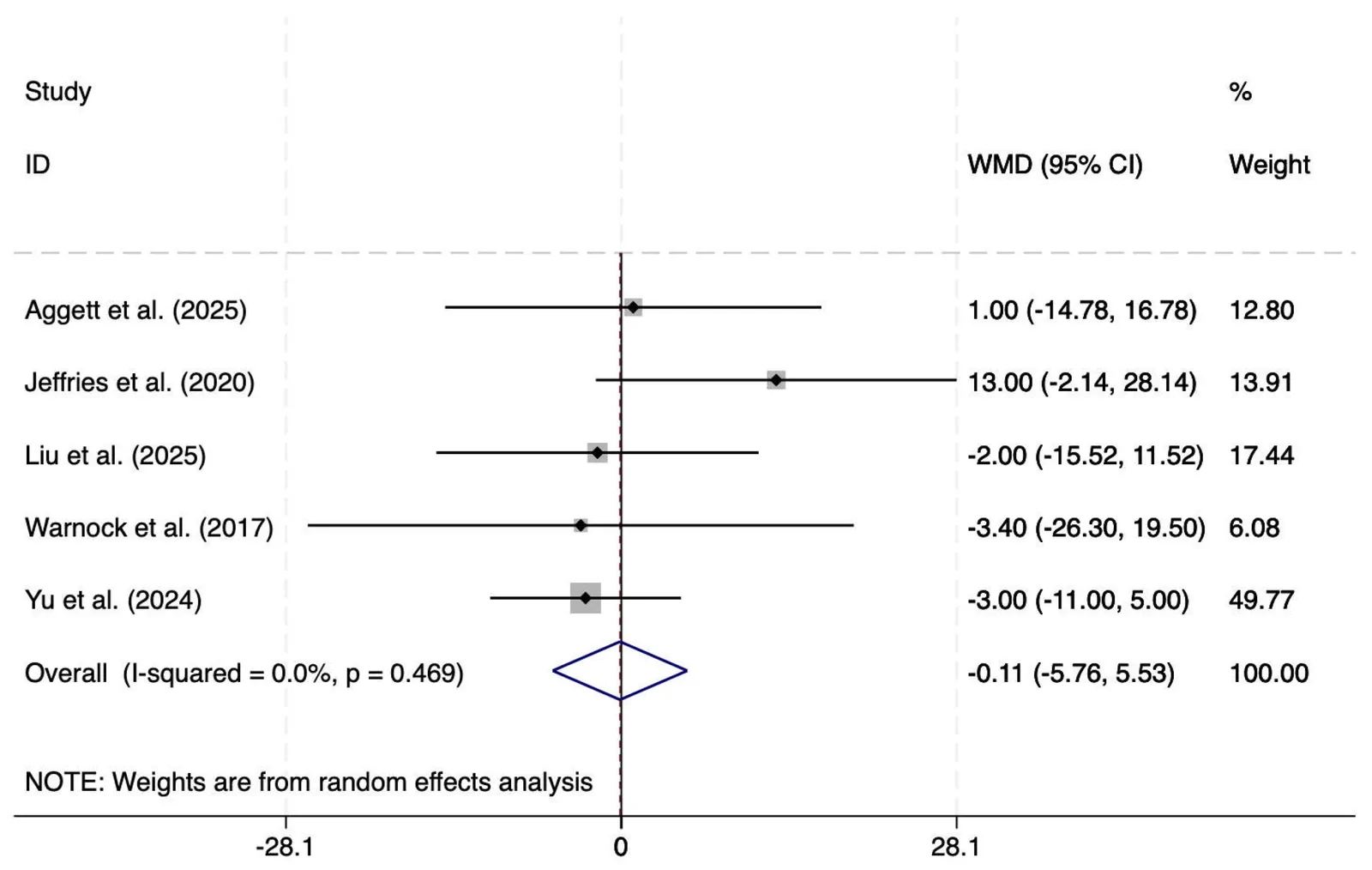
Effect of caffeine and taurine co-ingestion on heart rate. Data were obtained from Aggett et al. (2025) [14], Jeffries et al. (2020) [17], Liu et al. (2025) [19], Warnock et al. (2017) [23], and Yu et al. (2024) [24]. The solid vertical line at WMD = 0 represents the line of no effect, whereas the red dotted vertical line represents the pooled effect estimate from the random-effects model. Squares indicate study-specific estimates, with their sizes proportional to study weights; horizontal lines represent 95% CIs. The diamond represents the pooled WMD and its 95% CI. CI, confidence interval; HR, heart rate; WMD, weighted mean difference.

As shown in Figure 5, no significant difference was found between caffeine–taurine supplementation and placebo in HR response (WMD = −0.11, 95% CI: −5.76 to 5.53). Heterogeneity was negligible (*I*^2^ = 0%), and study-level effects were evenly distributed around the null line.

#### 3.4.4. Reaction Time (RT)

**Figure 6:**
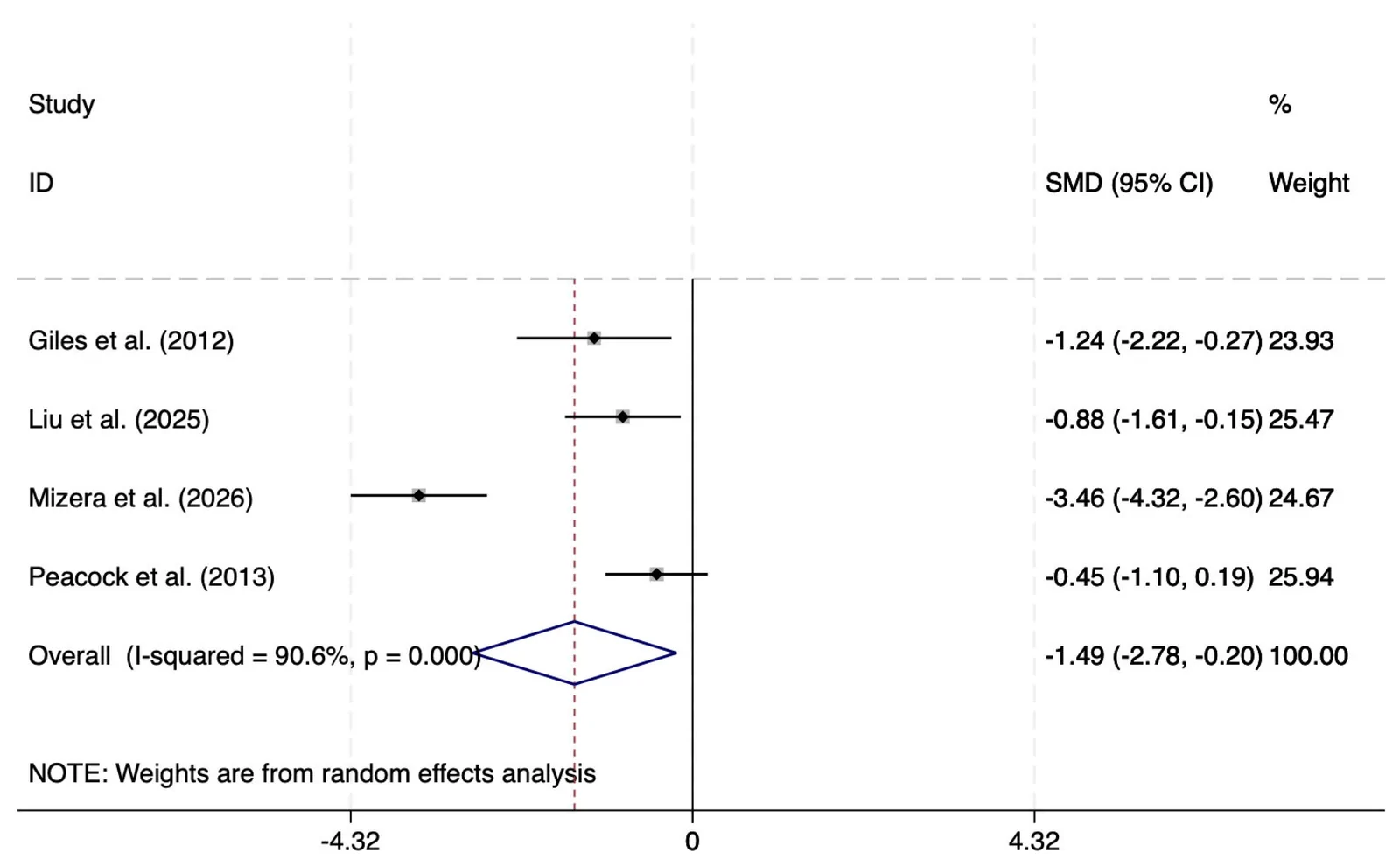
Effect of caffeine and taurine co-ingestion on reaction time. Data were obtained from Giles et al. (2012) [15], Liu et al. (2025) [19], Mizera et al. (2026) [20], and Peacock et al. (2013) [22]. The solid vertical line at SMD = 0 represents the line of no effect, whereas the red dotted vertical line represents the pooled effect estimate from the random-effects model. Negative SMD values indicate a shorter reaction time and favor caffeine–taurine co-ingestion. Squares indicate study-specific estimates, with their sizes proportional to study weights; horizontal lines represent 95% CIs. The diamond represents the pooled SMD and its 95% CI. CI, confidence interval; RT, reaction time; SMD, standardized mean difference.

Figure 6 shows the pooled analysis for RT. The pooled estimate favored a shorter reaction time (SMD = −1.49, 95% CI: −2.78 to −0.20), with a magnitude conventionally considered large. However, between-study heterogeneity was very high (*I*^2^ = 90.6%, τ^2^ = 1.56), indicating marked inconsistency across studies. The sensitivity analysis (Supplementary Figure S1) indicated that the pooled estimate was not driven by any single study. The 95% prediction interval ranged from −7.56 to 4.57 and crossed the null, suggesting that both the magnitude and direction of the effect may vary substantially across settings. Therefore, although the average pooled estimate favored supplementation, it should not be interpreted as evidence of a consistent improvement in reaction time.

Sensitivity analysis excluding Giles et al. showed that the improvement in reaction time remained significant (SMD = −0.64, 95% CI: −1.12 to −0.16; *I*^2^ = 0%; *p* = 0.010), indicating that the result was not driven by this study. Sensitivity analysis excluding Mizera et al. showed that the improvement in reaction time remained significant (SMD = −0.77, 95%CI: −1.19 to −0.34; *I*^2^ = 35.5%; *p* = 0.010), indicating that the result was not driven by this study.

#### 3.4.5. Rating of Perceived Exertion (RPE)

**Figure 7:**
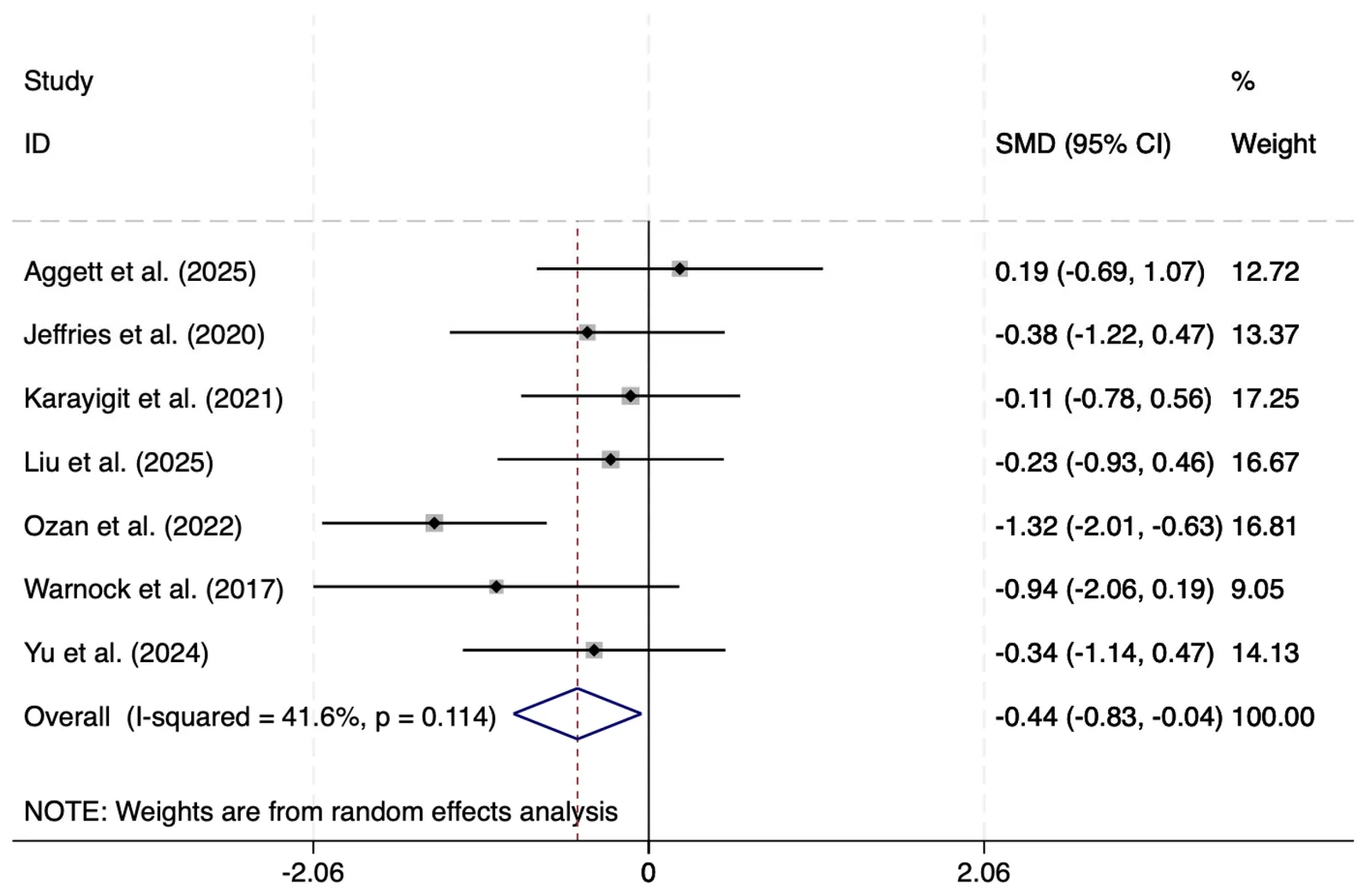
Effect of caffeine and taurine co-ingestion on rating of perceived exertion. Data were obtained from Aggett et al. (2025) [14], Jeffries et al. (2020) [17], Karayigit et al. (2021) [18], Liu et al. (2025) [19], Ozan et al. (2022) [21], Warnock et al. (2017) [23], and Yu et al. (2024) [24]. The solid vertical line at SMD = 0 represents the line of no effect, whereas the red dotted vertical line represents the pooled effect estimate from the random-effects model. Negative SMD values indicate a lower perceived exertion and favor caffeine–taurine co-ingestion. Squares indicate study-specific estimates, with their sizes proportional to study weights; horizontal lines represent 95% CIs. The diamond represents the pooled SMD and its 95% CI. CI, confidence interval; RPE, rating of perceived exertion; SMD, standardized mean difference.

As presented in Figure 7, caffeine–taurine supplementation significantly reduced perceived exertion compared with placebo (SMD = −0.44, 95% CI: −0.83 to −0.04). However, heterogeneity was substantial (*I*^2^ = 41.6%, τ^2^ = 0.12), suggesting variability across study designs and participant characteristics. The 95% prediction interval ranged from −1.45 to 0.58 and crossed the null. Thus, although the average estimate favored supplementation, the available evidence does not demonstrate a consistent reduction in perceived exertion across settings.

### 3.5. Subgroup Analysis Results

Exploratory subgroup results are presented in Supplementary Table S5. Because most strata contained only one or a small number of studies, these findings were considered descriptive and did not provide reliable evidence of effect modification.

### 3.6. Meta-Regression Results

As shown in Table 2, the exploratory meta-regression analyses did not reveal clear or consistent associations between the examined covariates and the pooled effects. However, the small number of studies and wide uncertainty around the estimates precluded reliable conclusions regarding potential moderation.

### 3.7. Publication Bias

Because each meta-analysis included fewer than 10 studies, funnel plots and statistical tests for small-study effects were not performed owing to their limited power and interpretability.

### 3.8. Sensitivity Analysis for Assumed Within-Participant Correlations

Under the primary assumption of r = 0.50 for crossover comparisons without recoverable paired information, caffeine–taurine supplementation was associated with longer TTE (SMD = 0.54, 95% CI: 0.03 to 1.05; *p* = 0.038; *I*^2^ = 16.0%), greater peak/maximal anaerobic power (SMD = 0.55, 95% CI: 0.21 to 0.89; *p* = 0.002; *I*^2^ = 0%), lower RPE (SMD = −0.44, 95% CI: −0.83 to −0.04; *p* = 0.029; *I*^2^ = 41.6%), and shorter RT (SMD = −1.49, 95% CI: −2.78 to −0.20; *p* = 0.024; *I*^2^ = 90.6%). No statistically detectable difference was observed for HR (WMD = −0.11 bpm, 95% CI: −5.76 to 5.53; *p* = 0.970; *I*^2^ = 0%).

Sensitivity analyses using r = 0.25 and r = 0.75 produced pooled estimates in the same direction for PP, RPE, and RT, and their confidence intervals continued to exclude the null. HR remained statistically inconclusive under both alternative assumptions. In contrast, the TTE result was sensitive to the assumed correlation: although the confidence interval excluded the null under the primary r = 0.50 assumption, it crossed the null when r = 0.25 (SMD = 0.436, 95% CI: −0.081 to 0.953; *p* = 0.098) and when r = 0.75 (SMD = 0.472, 95% CI: −0.008 to 0.951; *p* = 0.054). Heterogeneity did not increase monotonically with the assumed correlation. However, under the r = 0.75 assumption, *I*^2^ increased substantially for TTE, PP, RPE, and HR, whereas RT showed consistently considerable heterogeneity under all three assumptions (*I*^2^ = 90.6–93.1%). These findings indicate that the PP, RPE, and RT estimates were comparatively robust to the assumed within-participant correlation, whereas the statistical interpretation of TTE and the estimated heterogeneity for several outcomes remained sensitive to this assumption. Detailed results are presented in Supplementary Table S6.

## 4. Discussion

This study suggests that the combined intervention may be associated with small to moderate improvements in certain performance outcomes, although the magnitude and consistency of effects appear to vary across domains. The pooled estimates represent caffeine plus taurine versus placebo and should not be interpreted as evidence that taurine independently enhances performance or that the two ingredients act synergistically.

The most consistent signals were observed for time to exhaustion and PP. These findings may indicate a potential ergogenic tendency of caffeine–taurine co-supplementation in tasks requiring both endurance capacity and high-intensity output [25]. One possible explanation is that caffeine-related central nervous system stimulation could interact with taurine-related physiological roles in muscle function and calcium regulation [26,27]. However, because the pooled contrasts did not include direct comparisons with caffeine alone or taurine alone, they cannot establish whether taurine adds benefit, whether an interaction exists, or whether the observed effects are driven primarily or entirely by caffeine. The underlying mechanisms therefore remain speculative.

In contrast, no clear effect was observed on heart rate. This suggests that the supplementation is unlikely to meaningfully influence cardiovascular responses during exercise, and any performance-related effects may be mediated through perceptual or neuromuscular mechanisms rather than central hemodynamic changes [28]. This pattern is generally consistent with previous observations in caffeine-related literature, although direct comparisons remain limited [29].

For reaction time, the pooled standardized effect favored supplementation and was large in magnitude according to conventional thresholds. Nevertheless, this finding should be interpreted cautiously. Between-study heterogeneity was very high (*I*^2^ = 90.6%), and the wide 95% prediction interval (−7.56 to 4.57) included both potentially beneficial and unfavorable effects. Thus, the statistically significant average effect does not demonstrate a consistent or practically important improvement across settings. The certainty of evidence for this outcome was also rated very low. The observed variability may reflect differences in experimental design, reaction-time assessment protocols, intervention characteristics, and participant populations [30]. Meta-regression did not identify significant moderating effects, although the small number of available studies substantially limited the ability of these analyses to explain the heterogeneity.

For rating of perceived exertion, a reduction was observed, although the heterogeneity across studies was substantial. This indicates that perceptual responses to supplementation may be context-dependent [31]. Differences in exercise intensity, individual sensitivity to caffeine, and psychological factors may have contributed to the inconsistency. The meta-regression findings should not be interpreted as evidence of an absence of moderation. Owing to the limited number of studies, the analyses were underpowered and the estimated associations may be unstable. These exploratory findings require confirmation when additional studies become available.

Exploratory subgroup analyses did not provide reliable evidence that the effects differed according to training status, population type, or supplementation dose. Given the small number of studies within individual strata, any apparent patterns should be considered hypothesis-generating and should not be interpreted as evidence of effect modification without a statistically meaningful interaction. The available evidence was also insufficient to establish or exclude a dose–response relationship; therefore, the absence of a statistically detectable association should not be interpreted as evidence that no dose–response relationship exists.

Several additional limitations should be acknowledged. First, some included studies used commercial energy drinks or multi-ingredient formulations containing glucose, B vitamins, phosphatidylserine, or other potentially active components. Consequently, the pooled estimates reflect the effects of the tested beverage formulations and cannot be attributed exclusively to caffeine and taurine. Second, although the paired nature of the crossover trials was incorporated into the effect-size calculations, most studies did not report within-participant correlations or standard deviations of paired differences. An assumed within-participant correlation was therefore required for these studies, introducing uncertainty into the variance estimates and confidence intervals. Finally, the GRADE assessment indicated that the certainty of evidence ranged from very low to moderate across outcomes, mainly because of imprecision, inconsistency, indirectness, and risk-of-bias concerns. These limitations warrant cautious interpretation of the pooled findings.

Overall, the current evidence suggests that the caffeine–taurine intervention, considered as a combined package, may confer modest ergogenic benefits versus placebo, particularly for endurance and power-related performance. The certainty of this evidence remains limited, and no ingredient-specific or synergistic effect can be inferred.

## 5. Conclusions

Caffeine–taurine-only formulations were associated with a pooled difference favoring PP, although the magnitude was modest and the certainty of evidence remained limited. The expanded analysis, which included a commercial multi-ingredient energy drink, produced a smaller estimate; however, this product-level result cannot be attributed specifically to caffeine and taurine. Factorial trials including placebo, caffeine-only, taurine-only, and combined caffeine–taurine arms are required to determine ingredient-specific and interactive effects.

## Figures and Tables

**Table 1:** Characteristics of included studies.

Study	Year	Country	Sample Size	Sex	Age	Population	Design	Intervention Format	Additional Active Ingredients/Co-Interventions	Comparator Composition	CAF Dose	TAU Dose	Outcomes
Aggett et al. [14]	2025	UK	10	7 M/3 F	21 ± 1 years	Healthy, recreationally active adults	Double-blind, placebo-controlled, randomized crossover	Caffeine and taurine powders administered in matched gelatine capsules	None	Visually identical maltodextrin capsules	5 mg/kg	50 mg/kg	TTE, HR, RPE
Giles et al. [15]	2012	USA	48	18 M/30 F	20.08 ± 1.85 years	Undergraduate habitual caffeine consumers	Randomized, double-blind, mixed-factor repeated-measures design	Caffeine and taurine administered in identical capsules with a 500-mL beverage	Glucose (50 g) was administered in one randomized between-participant stratum; the other stratum received a matched artificially sweetened non-glucose drink. The beverage assignment was balanced across the caffeine/taurine conditions.	Capsules containing 0 mg caffeine and 0 mg taurine, together with the assigned glucose or matched non-glucose drink	200 mg	2000 mg	RT
Gwacham et al. [16]	2012	USA	20	20 M	19.7 ± 1.8 years	NCAA Division I football players	Double-blind, randomized crossover	Commercial powdered energy drink (AdvoCare Spark; one packet prepared in 237 mL water)	Multi-ingredient formulation containing carbohydrate, vitamins, minerals, amino acids, and other potentially active constituents	Citrus-flavored water containing maltodextrin; matched for volume and energy content	120 mg	200 mg	PP
Jeffries et al. [17]	2020	UK	11	11 M	21 ± 2 years	Physically active university men accustomed to repeated-sprint exercise	Randomized, double-blind crossover with repeated measures	Caffeine and taurine powders administered in gelatine capsules	None	1080 mg maltodextrin in matched capsules	80 mg	1000 mg	RPE, PP, HR
Karayigit et al. [18]	2021	Turkey	17	17 F	23.4 ± 2.1 years	Female team-sport athletes habituated to caffeine	Double-blind, randomized crossover	Caffeine and taurine powders administered in indistinguishable gelatine capsules	None	300 mg maltodextrin in matched capsules	6 mg/kg	1000 mg	RPE, PP
Liu et al. [19]	2025	China	16	16 M	23.69 ± 2.15 years	Male university football players	Double-blind, randomized crossover	Caffeine and taurine powders administered in gelatine capsules	None	5 mg/kg maltodextrin in matched capsules	5 mg/kg	50 mg/kg	TTE, RPE, PP, HR, RT
Mizera et al. [20]	2026	Poland	54	54 M	19–32 years (range)	Professional male football players	Randomized, placebo-controlled, single-blind, parallel-group trial	Identical capsules administered for 10 days; only the taurine-plus-caffeine (TC) arm was included in the meta-analysis	None in the analyzed TC arm. The separate TCP arm containing phosphatidylserine (300 mg/day) was not included in this comparison.	Lactose capsules	200 mg	1500 mg	RT
Ozan et al. [21]	2022	Turkey	20	20 M	22.14 ± 1.42 years	Elite male boxers	Double-blind, randomized crossover	Caffeine and taurine powders administered in indistinguishable gelatine capsules	None	300 mg maltodextrin in matched capsules	6 mg/kg	3000 mg	RPE, PP
Peacock et al. [22]	2013	Australia	19	19 F	20.8 ± 0.9 years	Female undergraduate students	Double-blind, placebo-controlled, within-subject crossover	Caffeine and taurine administered in identical opaque capsules	None; corn flour was used only to match capsule weight	Matched corn flour-containing placebo capsules	80 mg	1000 mg	RT
Warnock et al. [23]	2017	UK	7	7 M	20.8 ± 0.9 years	Male university team-sport players	Randomized, single-blind crossover	Caffeine and taurine powders administered in gelatine capsules	None	5 mg/kg maltodextrin in matched capsules	5 mg/kg	50 mg/kg	RPE, PP, HR
Yu et al. [24]	2024	China	12	NR	23.75 ± 2.41 years	University students majoring in sport/physical education	Randomized, single-blind crossover	Caffeine and taurine powders administered in gelatine capsules	None	5 mg/kg maltodextrin in matched capsules	5 mg/kg	50 mg/kg	TTE, RPE, HR

CAF, caffeine; TAU, taurine; TC, taurine plus caffeine; TCP, taurine plus caffeine plus phosphatidylserine; DB, double-blind; SB, single-blind; RCT, randomized controlled trial; MD, maltodextrin; M, male; F, female; NR, not reported; TTE, time to exhaustion; HR, heart rate; RPE, rating of perceived exertion; PP, peak/maximal anaerobic power; RT, reaction time.

**Table 2:** Meta-regression analysis.

Outcome	Variable	Beta	SE	*p*-Value
Rating of Perceived Exertion	year	0.119	0.153	0.517
Rating of Perceived Exertion	Sex	−0.656	0.703	0.449
Rating of Perceived Exertion	population type	−0.111	0.580	0.865
Rating of Perceived Exertion	dose level	−0.163	1.124	0.897
reaction time	year	−0.591	1.516	0.764
reaction time	Sex	−0.295	0.758	0.764
reaction time	population type	−0.857	0.892	0.513
reaction time	dose level	−0.295	0.758	0.764

These analyses are exploratory and hypothesis-generating. Results should be interpreted cautiously because of the limited number of studies per covariate. β coefficients and standard errors are reported to three decimal places. *p* values are reported to three decimal places or as *p* < 0.001.

## Data Availability

The data supporting the findings of this study are included in the article and its Supplementary Materials. Further inquiries may be directed to the corresponding author.

## Supplementary Materials

The following supporting information can be downloaded at: https://www.mdpi.com/article/10.3390/nu18203434/s1, Table S1: Search strategy for each database; Table S2: Full-text reports excluded after eligibility assessment and specific reasons for exclusion; Table S3: Washout, Administration Timing, Habitual Caffeine Exposure, and Exercise/Testing Protocols; Table S4: GRADE assessment and summary of findings for caffeine–taurine beverage supplementation versus placebo; Table S5: Subgroup analysis; Table S6: Sensitivity analysis for assumed within-participant correlations; Table S7: Study-specific RoB 2 judgments and supporting justifications; Table S8: Study-specific definitions and measurement characteristics of peak/maximal anaerobic power outcomes included in the meta-analysis; Figure S1: Sensitivity analysis of reaction time; Figure S2: Expanded secondary product-level analysis of peak/maximal anaerobic power including Gwacham et al., which evaluated a commercial caffeine–taurine-containing multi-ingredient energy drink; File S1: Prisma checklist.

## Abbreviations

The following abbreviations are used in this manuscript:

Abbreviation	Definition
CAF	Caffeine
CI	Confidence interval
DB	Double-blind
F	Female
HR	Heart rate
M	Male
NA	Not applicable
PP	Peak/maximal anaerobic power
PRISMA	Preferred Reporting Items for Systematic Reviews and Meta-Analyses
RCT	Randomized controlled trial
RoB 2	Revised Cochrane risk-of-bias tool for randomized trials
