# Supplementary material for: Effects of Caffeine–Taurine Co-Supplementation on Exercise Performance: A Systematic Review and Meta-Analysis
Source: Nutrients. 2026 Oct 20;18(20):3434. doi: 10.3390/nu18203434 (PMC13708174; doi:10.3390/nu18203434)
Supplement: Supplementary file 1 [file nutrients-18-03434-s001.zip › nutrients-4466371-supplementary.pdf]

Supplementary

Table S1 *Search strategy for each database*

pubmed	*((("Caffeine"[Mesh] OR caffein*[Title/Abstract] OR "1,3,7-trimethylxanthine"[Title/Abstract] OR trimethylxanthine*[Title/Abstract]) AND ("Taurine"[Mesh] OR taurin*[Title/Abstract] OR "2-aminoethanesulfonic acid"[Title/Abstract] OR "2-aminoethane sulfonic acid"[Title/Abstract])) OR ("Energy Drinks"[Mesh] OR "energy drink"[Title/Abstract] OR "energy drinks"[Title/Abstract] OR "energy beverage"[Title/Abstract] OR "energy beverages"[Title/Abstract] OR "caffeinated drink"[Title/Abstract] OR "caffeinated drinks"[Title/Abstract] OR "caffeinated beverage"[Title/Abstract] OR "caffeinated beverages"[Title/Abstract] OR "Red Bull"[Title/Abstract])) AND ("Exercise"[Mesh] OR "Exercise Test"[Mesh] OR "Physical Functional Performance"[Mesh] OR "Athletic Performance"[Mesh] OR "Sports"[Mesh] OR "Cognition"[Mesh] OR "Memory"[Mesh] OR "Attention"[Mesh] OR "Reaction Time"[Mesh] OR "Psychomotor Performance"[Mesh] OR "Fatigue"[Mesh] OR "Heart Rate"[Mesh] OR exercis*[Title/Abstract] OR sport*[Title/Abstract] OR athlet*[Title/Abstract] OR perform*[Title/Abstract] OR ergogenic*[Title/Abstract] OR endurance[Title/Abstract] OR "time to exhaustion"[Title/Abstract] OR sprint*[Title/Abstract] OR cycl*[Title/Abstract] OR Wingate[Title/Abstract] OR power[Title/Abstract] OR fatigue[Title/Abstract] OR "perceived exertion"[Title/Abstract] OR cognit*[Title/Abstract] OR memory[Title/Abstract] OR attention[Title/Abstract] OR "reaction time"[Title/Abstract] OR psychomotor[Title/Abstract] OR "heart rate"[Title/Abstract]) AND (randomized controlled trial[Publication Type] OR controlled clinical trial[Publication Type] OR random*[Title/Abstract] OR placebo*[Title/Abstract] OR trial*[Title/Abstract] OR crossover*[Title/Abstract] OR "cross-over"[Title/Abstract] OR "cross over"[Title/Abstract] OR blind*[Title/Abstract])*
Embase	*((('caffeine'/exp OR caffein*:ti,ab,kw OR '1,3,7-trimethylxanthine':ti,ab,kw OR trimethylxanthine*:ti,ab,kw) AND ('taurine'/exp OR taurin*:ti,ab,kw OR '2-aminoethanesulfonic acid':ti,ab,kw OR '2-aminoethane sulfonic acid':ti,ab,kw)) OR ('energy drink'/exp OR 'energy drink':ti,ab,kw OR 'energy drinks':ti,ab,kw OR 'energy beverage':ti,ab,kw OR 'energy beverages':ti,ab,kw OR 'caffeinated drink':ti,ab,kw OR 'caffeinated drinks':ti,ab,kw OR 'caffeinated beverage':ti,ab,kw OR 'caffeinated beverages':ti,ab,kw OR 'red bull':ti,ab,kw)) AND ('exercise'/exp OR 'cognition'/exp OR 'memory'/exp OR 'reaction time'/exp OR 'heart rate'/exp OR exercis*:ti,ab,kw OR sport*:ti,ab,kw OR athlet*:ti,ab,kw OR perform*:ti,ab,kw OR ergogenic*:ti,ab,kw OR endurance:ti,ab,kw OR 'time to exhaustion':ti,ab,kw OR sprint*:ti,ab,kw OR cycl*:ti,ab,kw OR wingate:ti,ab,kw OR power:ti,ab,kw OR fatigue:ti,ab,kw OR 'perceived exertion':ti,ab,kw OR cognit*:ti,ab,kw OR memory:ti,ab,kw OR attention:ti,ab,kw OR 'reaction time':ti,ab,kw OR psychomotor:ti,ab,kw OR 'heart rate':ti,ab,kw) AND ('randomized controlled trial'/exp OR 'controlled clinical trial'/exp OR 'crossover procedure'/exp OR 'double blind procedure'/exp OR 'single blind procedure'/exp OR random*:ti,ab,kw OR placebo*:ti,ab,kw OR trial*:ti,ab,kw OR crossover*:ti,ab,kw OR 'cross-over':ti,ab,kw OR 'cross over':ti,ab,kw OR blind*:ti,ab,kw)*
Cochenae	*#1 MeSH descriptor: [Caffeine] explode all trees*<br><br>*#2 MeSH descriptor: [Taurine] explode all trees*

	#3 MeSH descriptor: [Energy Drinks] explode all trees<br><br>*#4 (caffein*:ti,ab,kw AND (taurin*:ti,ab,kw OR "2-aminoethanesulfonic acid":ti,ab,kw OR "2-aminoethane sulfonic acid":ti,ab,kw))*<br><br>*#5 (((energy OR caffeinated) NEAR/1 (drink* OR beverage*)) OR "Red Bull"):ti,ab,kw*<br><br>*#6 (#1 AND #2) OR #3 OR #4 OR #5*<br><br>*#7 MeSH descriptor: [Exercise] explode all trees*<br><br>*#8 MeSH descriptor: [Exercise Test] explode all trees*<br><br>*#9 MeSH descriptor: [Physical Functional Performance] explode all trees*<br><br>*#10 MeSH descriptor: [Athletic Performance] explode all trees*<br><br>*#11 MeSH descriptor: [Sports] explode all trees*<br><br>*#12 MeSH descriptor: [Cognition] explode all trees*<br><br>*#13 MeSH descriptor: [Memory] explode all trees*<br><br>*#14 MeSH descriptor: [Attention] explode all trees*<br><br>*#15 MeSH descriptor: [Reaction Time] explode all trees*<br><br>*#16 MeSH descriptor: [Psychomotor Performance] explode all trees*<br><br>*#17 MeSH descriptor: [Fatigue] explode all trees*<br><br>*#18 MeSH descriptor: [Heart Rate] explode all trees*<br><br>*#19 (exercis* OR sport* OR athlet* OR perform* OR ergogenic* OR endurance OR "time to exhaustion" OR sprint* OR cycl* OR Wingate OR power OR fatigue OR "perceived exertion" OR cognit* OR memory OR attention OR "reaction time" OR psychomotor OR "heart rate"):ti,ab,kw*<br><br>*#20 #7 OR #8 OR #9 OR #10 OR #11 OR #12 OR #13 OR #14 OR #15 OR #16 OR #17 OR #18 OR #19*<br><br>*#21 #6 AND #20*
Web of science	*TS=((((caffein* AND (taurin* OR "2-aminoethanesulfonic acid" OR "2-aminoethane sulfonic acid")) OR "energy drink*" OR "energy beverage*" OR "caffeinated drink*" OR "caffeinated beverage*" OR "Red Bull") AND (exercis* OR sport* OR athlet* OR perform* OR ergogenic**

	OR endurance OR "time to exhaustion" OR sprint OR cycl* OR Wingate OR power OR fatigue OR "perceived exertion" OR cognit* OR memory OR attention OR "reaction time" OR psychomotor OR "heart rate") AND (random* OR placebo* OR trial* OR crossover* OR "cross-over" OR "cross over" OR blind*)))*

Table S2 *Full-text reports excluded after eligibility assessment and specific reasons for exclusion.*

Excluded report	Full citation	Specific reason for exclusion
Warburton et al. (2001)	Warburton DM, Bersellini E, Sweeney E. An evaluation of a caffeinated taurine drink on mood, memory and information processing in healthy volunteers without caffeine abstinence. Psychopharmacology (Berl). 2001;158(3):322–328. doi:10.1007/s002130100884.	No relevant prespecified exercise-performance outcome.
Bichler et al. (2006)	Bichler A, Swenson A, Harris MA. A combination of caffeine and taurine has no effect on short term memory but induces changes in heart rate and mean arterial blood pressure. Amino Acids. 2006;31(4):471–476. doi:10.1007/s00726-005-0302-x.	No relevant outcome

Table S3 *Washout, Administration Timing, Habitual Caffeine Exposure, and Exercise/Testing Protocols*

Study	Year	Washout / visit spacing	Administration-to-test interval	Habitual caffeine use / pretrial restrictions	Detailed exercise/testing protocol
Aggett et al.	2025	Experimental visits separated by 72–96 h	60 min before exercise	7 habituated and 3 non-habituated (>100 mg/day criterion); caffeine abstinence not explicitly reported. No alcohol or other supplements for 24 h and no strenuous exercise for 48 h before trials.	In 35°C and 40% RH, constant-load cycling at the thermoneutral gas-exchange-threshold power (59 ± 4% VO2peak; 148 ± 42 W), 70 rpm, until exhaustion or Tcore reached 39.5°C. Preliminary ramp test: 5 min at 70 W, 5 min rest, then 70 W with 21 W/min increments.
Giles et al.	2012	3 days between treatment days; additionally, 24 h caffeine abstinence before	Attention Network Task at 30 min; N-back and reaction-time tasks at approximately 60 min	≥200 mg/day; mean 527.59 ± 277.13 mg/day. Abstained from caffeine, over-the-counter medications, and herbal supplements for 24 h before each session.	No exercise protocol. Cognitive battery: Attention Network Task (3 × 96 trials); verbal, spatial, and object N-back tasks at 1-, 2-, and 3-back loads; and simple and two-choice reaction-time tasks (5 blocks each). Mood, withdrawal symptoms,

		each session			salivary cortisol, and HR were also assessed.
Gwacham and Wagner	2012	7 days between trials	Approximately 60 min before the Running-based Anaerobic Sprint Test	Previous-month caffeinated-beverage use assessed with a self-reported caffeine score; mg/day and category counts NR. Participants avoided alcohol, nicotine, and other stimulants on the day before testing; caffeine abstinence was not explicitly stated.	After a standardized breakfast and 25 min dynamic warm-up, participants completed 6 × 35 m maximal sprints on artificial turf with 10 s recovery. Sprint time, anaerobic power, and fatigue index were calculated.
Jeffries et al.	2020	1 week between visits	60 min before repeated-sprint testing	A mix of regular caffeine users; quantitative intake NR. Avoided alcohol and caffeinated products for 24 h, strenuous exercise for 48 h, and food for 3 h before testing.	Cycle ergometer: 5 min at 100 W and 80 rpm; two 6 s practice sprints (70% and 100% effort) separated by 1 min; 2 min rest; then 10 × 6 s maximal seated sprints separated by 24 s complete static rest. RPE and HR were recorded after each sprint; lactate at baseline and after sprints 5 and 10.
Karayigit et al.	2021	At least 48 h between trials	60 min before the Wingate test	340.1 ± 28.6 mg/day; inclusion required 3–6 mg/kg/day during the previous 3 months. Avoided caffeine and vigorous exercise for 24 h before testing.	5 min cycle warm-up at 60 W with unloaded 5 s sprints during minutes 2 and 3; then one 30 s maximal Wingate from a stationary start against 7.5% BM. Peak and mean power, fatigue index, HR, lactate, and postexercise RPE were assessed.
Liu and Rong	2025	Authors reported that each visit was separated by no more than 3 days and described this as sufficient washout	Supplement 60 min before baseline measurements; warm-up and exercise testing followed	No chronic caffeine-use habits; quantitative intake NR. Participants avoided listed caffeine- and taurine-containing dietary sources throughout the testing period and did not eat for 2 h before testing.	Simulated altitude of 2500 m (15.3% O2). Warm-up: 5 min at 70 rpm/60 W plus three 3 s maximal sprints; CMJ and Stroop; 5 min hypoxic wash-in; incremental cycling increased 30 W/min to exhaustion at 70 rpm; 15 min recovery with Stroop; then 6 × 10 s maximal sprints against 7.5% BM with 50 s recovery.
Mizera et al.	2026	N/A (parallel-	Final assigned	Baseline habitual caffeine intake NR.	Standardized 105 min football session: 15 min

		group study)	dose 60 min before the standardized training session	Participants refrained from caffeine and dietary supplements for 7 days before the study; regular supplement users were excluded.	warm-up, 15 min technical drills, 30 min match simulation, 15 min drills, and a final 30 min match simulation. Outcomes included 30 m sprint, visual and auditory RT, GPS-derived workload, and technical/tactical measures.
Ozan et al.	2022	NR as an explicit washout; the authors stated that the four sessions occurred "in a 72 h window" at the same time of day	60 min before the Wingate test	Quantitative habitual intake NR. Caffeine and other stimulants were restricted for 48 h before each experimental session.	5 min cycle warm-up at 60 W with unloaded 5 s sprints during minutes 2 and 3; one 30 s maximal Wingate against 7.5% BM. RPE and lactate immediately after; balance at approximately 1 min, Illinois agility at approximately 3 min, and Stroop testing at approximately 3.5 min after Wingate.
Peacock et al.	2013	Minimum 2 days and maximum 7 days between sessions	Cognitive tasks commenced 45 min after administration	Inclusion required ≥50 mg/day; mean 228 mg/day (range 50–405). Before sessions: no food for 4 h, no caffeine or energy drinks for 8 h, no alcohol for 24 h, and no psychoactive substances for 48 h.	Visual oddball task: safe/accident images at an 8:2 ratio, 200 trials, 250 ms presentation, 1000 ms maximum response window. Stimulus-degradation task: digits 2, 4, and 5 at three degradation levels, 336 trials, 400 ms presentation, 1400 ms maximum response window.
Warnock et al.	2017	48 h between experimental trials	60 min seated rest after ingestion before baseline measurements and exercise testing	Quantitative habitual intake NR. Avoided caffeine- and taurine-containing food/drinks for 24 h, food for 2 h, and strenuous exercise for 48 h before testing.	Cycle ergometer at 0.075 kg/kg BM: 5 min warm-up at 0.9 W/kg and 80 rpm including a 5 s sprint, followed by 3 min passive recovery; then 3 × 30 s Wingate tests separated by 2 min active recovery at 0.9 W/kg and 65 rpm. HR/RPE and lactate were recorded.
Yu et al.	2024	7 days between conditions	60 min before testing	Non-caffeine-habituated; quantitative intake NR. Avoided caffeine- and taurine-	In 35 ± 1°C and 65 ± 2% RH: 5 min warm-up at 100 W, 3 min preparation, then constant-load cycling at ventilatory-threshold

	containing sources for 1 week before the first trial and throughout testing; diet controlled for 24 h and strenuous exercise avoided for 48 h before testing.	power until exhaustion. CMJ was assessed at baseline, immediately after exercise, and 3 min postexercise; HR, Tcore, RPE, and thermal sensation were recorded repeatedly.

***Table S4 GRADE** assessment and summary of findings for caffeine–taurine beverage supplementation versus placebo*

Outcome	Risk of bias	Inconsistency	Indirectness	Imprecision	Publication bias	Pooled effect (95% CI)	Certainty
**Time to exhaustion (TTE)**	Not serious	Not serious	Serious (a)	Serious (c)	Not detected; uncertain (i)	SMD 0.54 (0.03 to 1.05)	**Low** ⊕⊕◯◯
**Peak/maximal anaerobic power (PP)**	Not serious	Not serious	Serious (b)	Serious (c)	Not detected; uncertain (i)	SMD 0.55 (95% CI 0.21 to 0.89)	**Low** ⊕⊕◯◯
**Heart rate (HR)**	Not serious	Not serious	Not serious	Serious (d)	Not assessed reliably (i)	WMD −0.11 bpm (−5.76 to 5.53)	**Moderate** ⊕⊕⊕◯
**Rating of perceived exertion (RPE)**	Not serious	Not serious	Not serious	Serious (c)	Not detected; uncertain (i)	SMD −0.44 (−0.83 to −0.04)	**Moderate** ⊕⊕⊕◯
**Reaction time (RT)**	Serious (e)	Serious (f)	Serious (g)	Serious (h)	Not detected; uncertain (i)	SMD −1.49 (−2.78 to −0.20)	**Very low** ⊕◯◯◯

Table S5. *Subgroup analysis.*

Outcome	Subgroup Type	Subgroup	Effect (95% CI)	I2 (%)	No. of Studies
HR(WMD)	Population	Healthy adults	1.00 (-14.78, 16.78)	NA	2
	Population	Trained athletes	7.14 (-8.26, 22.54)	27.1	2
	Population	Students	-2.74 (-9.63, 4.15)	0.0	1
	Sex	Male	0.15 (-6.80, 7.11)	15.1	3
	Sex	Mixed	1.00 (-14.78, 16.78)	NA	1
	Dose	Moderate	0.15 (-6.80, 7.11)	15.1	3
	Dose	High	1.00 (-14.78, 16.78)	NA	1
PP (SMD)	Population	Trained athletes	0.40 (0.06, 0.74)	0	4
	Population	Students	0.78 (0.06, 1.50)	0	1
	Population	Healthy adults	0.62 (-0.46, 1.70)	NA	1
	Sex	Male	0.72 (0.17, 1.27)	0	4
	Sex	Female	0.52 (-0.17, 1.20)	NA	1
	Dose	Low	0.25 (-0.37, 0.87)	NA	1
	Dose	Moderate	0.02 (-0.40, 0.44)	2.7	4
RT(SMD)	Population	Healthy adults	-0.32 (-1.42, 0.78)	NA	1
	Population	Trained athletes	-0.61 (-1.37, 0.15)	68.9	3
	Population	Students	-0.28 (-0.80, 0.25)	NA	1
	Sex	Male	-0.59 (-1.11, 0.06)	49.0	4
	Sex	Female	-0.11 (-0.78, 0.56)	NA	1
	Sex	Mixed	-0.32 (-1.42, 0.78)	58.3	2
	Dose	High	-2.36 (-4.53, -0.19)	91.0	2
	Dose	Moderate	-0.88 (-1.61, -0.15)	NA	1
	Dose	Low	-0.45 (-1.10, 0.19)	NA	1
RPE(SMD)	Population	Healthy adults	-0.32 (-1.42, 0.78)	58.3	2
	Population	Trained athletes	-2.16 (-4.68, 0.37)	95.0	2
	Population	Students	-0.76 (-1.51, -0.01)	42.7	2
	Sex	Male	-2.16 (-4.68, 0.37)	95.0	2
	Sex	Female	-0.45 (-1.10, 0.19)	NA	1
	Sex	Mixed	-1.24 (-2.22, -0.27)	NA	1
	Dose	Moderate	-0.53 (-0.93, -0.12)	37.7	1
	Dose	High	0.19 (-0.69, 1.07)	NA	1
TTE(SMD)	Population	Healthy adults	0.62 (-0.46, 1.70)	NA	1
	Population	Trained athletes	0.40 (0.06, 0.74)	0	1
	Population	Students	0.78 (0.06, 1.50)	0	1
	Sex	Male	0.72 (0.17, 1.27)	0.3	2
	Sex	Mixed	0.10 (-0.78, 0.98)	NA	1
	Dose	Low	0.25 (-0.37, 0.87)	NA	1
	Dose	Moderate	0.55 (0.21, 0.89)	0.7	1
	Dose	High	0.10 (-0.78, 0.98)	NA	1

This analysis is exploratory. Estimates based on one study represent individual study effects rather than pooled subgroup effects. I^2 is not applicable to single-study strata, and no conclusions regarding subgroup differences should be drawn from these estimates.

Table S6 Sensitivity analysis for assumed within-participant correlations

Outcome	(r=0.25): effect (95% CI); P; I^2	(r = 0.50), primary: effect (95% CI); P; I^2	(r=0.75): effect (95% CI); P; I^2
TTE, k=3	0.436 (−0.081, 0.953); .098; 59.0%	0.54 (0.03, 1.05); .038; 16.0%	0.472 (−0.008, 0.951); .054; 72.8%
PP, k=5	0.408 (0.191, 0.626); <.001; 7.0%	0.55 (0.21, 0.89); .002; 0.0%	0.404 (0.143, 0.666); .002; 63.8%
RPE, k=7	−0.412 (−0.780, −0.044); .028; 49.0%	−0.44 (−0.83, −0.04); .029; 41.6%	−0.390 (−0.711, −0.070); .017; 72.3%
RT, k=4	−1.458 (−2.478, −0.438); .005; 91.4%	−1.49 (−2.78, −0.20); .024; 90.6%	−1.412 (−2.252, −0.572); .001; 93.1%
HR, k=5	0.273 (−5.314, 5.860); .924; 15.1%	−0.11 (−5.76, 5.53); .970; 0.0%	1.029 (−4.766, 6.824); .728; 70.2%

Table S7 Study-specific RoB 2 judgments and supporting justifications.

Study	D1	D2	D3	D4	D5	Domain S	Overall
Aggett 2025	Low: balanced AB/BA sequence and opaque sealed allocation	Low: double-blind, identical capsules	Low: relevant data complete	Low: objective or standardized blinded measurements	Low: specified outcomes reported	Low: 72–96 h and balanced sequence	Low
Giles 2012	Low: Latin-square counterbalancing	Some concerns: blinding implementation and success insufficiently reported	Low	Some concerns for subjective outcomes; computerized RT was objective	Low	Low: 3-day spacing and Latin-square design	Some concerns
Gwacham 2012	Some concerns: sequence generation and concealment not reported	Low: double-blind matched placebo	Low	Low: automated sprint and power measurements	Low	Some concerns: 7-day interval but sequence balance and carryover testing not reported	Some concerns
Jeffries 2020	Low: random-number allocation	Low: double-blind matched capsules	Low	Low: objective outcomes and standardized	Low	Some concerns: 1-week interval but sequence balance and formal carryover	Some conc

				RPE under blinding		testing not reported	erns
Karayigit 2021	Low: randomized crossover design	Low: double-blind indistinguishable capsules	Low	Low: standardized RPE and objective performance measures	Low	Some concerns: ≥48 h but sequence balance and carryover testing not reported	Some concerns
Liu 2025	Low: computer-generated allocation	Low: double-blind procedures	Low	Low: instrument-derived and standardized outcomes	Low	Some concerns: no minimum washout interval reported	Some concerns
Mizera 2026	Some concerns: baseline comparability of eligible groups incompletely reported	High: single-blind design could influence delivery or encouragement	Low	Some concerns for coach-rated outcomes; objective RT was instrument-derived	Some concerns: retrospective registration and no prospective analysis plan	N/A: parallel design	High
Ozan 2022	Low: randomized crossover design	Low: double-blind indistinguishable capsules	Low	Low: standardized blinded RPE assessment	Low	Some concerns: four sessions within 72 h without explicit washout	Some concerns
Peacock 2013	Low: explicitly counterbalanced allocation	Low: participants, assessors and analysts blinded	Low	Low: computerized RT measurement	Low	Low: counterbalancing and 2–7-day spacing	Low
Warnock 2017	Low: randomized crossover design	Low: participant blinding and standardized procedures	Low	Low: objective outcomes and standardized RPE	Low	Some concerns: 48 h and no order effect, but no formal carryover analysis	Some concerns

Yu 2024	Low: randomized balanced sequences	Low: blinded standardized administration	Low	Low: objective and standardized blinded outcomes	Low	Low: balanced sequences and 7-day interval	Low

Table S8 Study-specific definitions and measurement characteristics of peak/maximal anaerobic power outcomes included in the meta-analysis.

Study	Extracted variable	Protocol	Unit	Time point	Analysis
Jeffries et al. (2020)	Average peak power across 10 sprints	10 × 6-s maximal cycling sprints; 24-s recovery	W	Test beginning 60 min after ingestion	Primary
Karayigit et al. (2021)	Peak power	Single 30-s Wingate test	W	60 min after ingestion	Primary
Liu et al. (2025)	Peak power across six sprints	6 × 10-s maximal cycling sprints; 50-s recovery	W	Following the exhaustion test and 15-min recovery	Primary
Ozan et al. (2022)	Peak power	Single 30-s Wingate test	W/kg	60 min after ingestion	Primary
Warnock et al. (2017)	Mean peak power across three Wingate tests	3 × 30-s Wingate tests; 2-min recovery	W/kg	Test beginning 60 min after ingestion	Primary
Gwacham et al. (2012)	RAST-derived anaerobic power	6 × 35-m maximal running sprints; 10-s recovery	W	Approximately 60 min after ingestion	Expanded secondary

Abbreviations: W, watts; W/kg, watts per kilogram. Peak power refers to the highest power attained within a sprint, whereas mean peak power refers to the average of sprint-specific peak-power values across repeated bouts. Mean power over the complete sprint duration, minimum power, sprint time, total work, and fatigue indices were not pooled. Gwacham 2012 was included only in the expanded secondary analysis because it evaluated a commercial multi-ingredient product and used a running-based protocol.

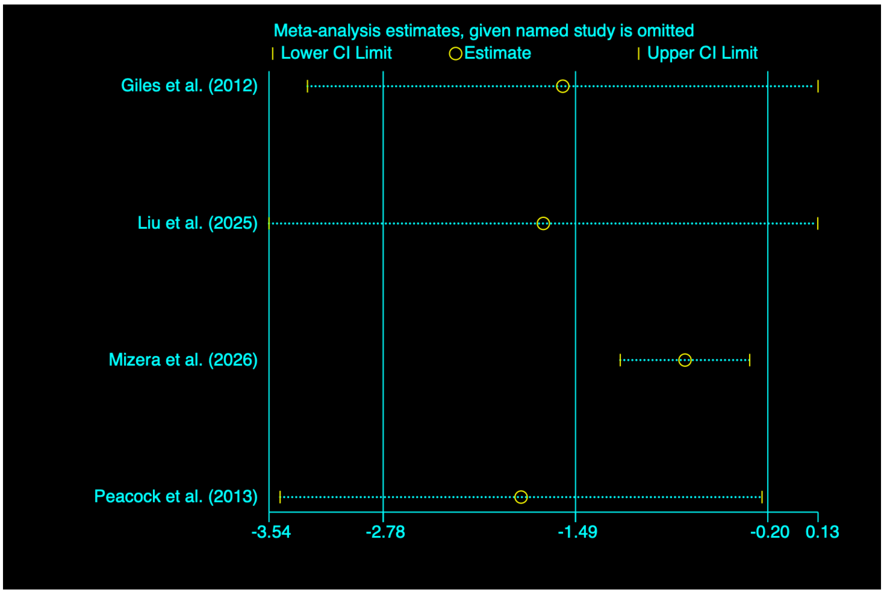

Figure S1. Sensitivity analysis of reaction time

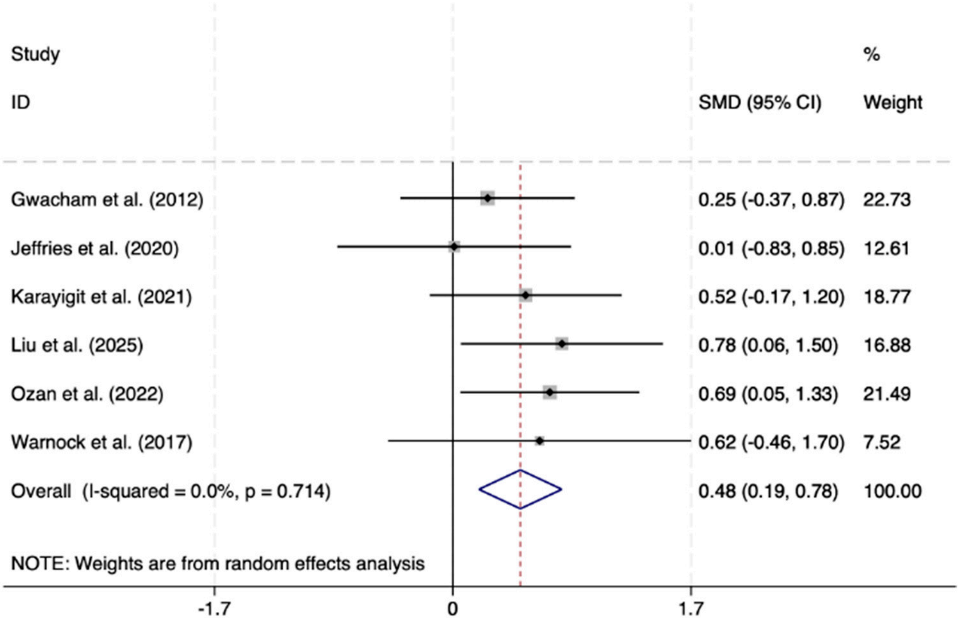

Figure S2. Expanded secondary product-level analysis of peak/maximal anaerobic power including Gwacham et al., which evaluated a commercial caffeine–taurine-containing multi-ingredient energy drink.

File S1 *Prisma checklist*

Section and Topic	Item #	Checklist item	Location where item is reported
**TITLE**			
Title	1	Identify the report as a systematic review.	p. 1
**ABSTRACT**			
Abstract	2	See the PRISMA 2020 for Abstracts checklist.	p. 1
**INTRODUCTION**			
Rationale	3	Describe the rationale for the review in the context of existing knowledge.	pp. 1–2
Objectives	4	Provide an explicit statement of the objective(s) or question(s) the review addresses.	p. 2
**METHODS**			

Section and Topic	Item #	Checklist item	Location where item is reported
Eligibility criteria	5	Specify the inclusion and exclusion criteria for the review and how studies were grouped for the syntheses.	pp. 2–3
Information sources	6	Specify all databases, registers, websites, organisations, reference lists and other sources searched or consulted to identify studies. Specify the date when each source was last searched or consulted.	p. 2
Search strategy	7	Present the full search strategies for all databases, registers and websites, including any filters and limits used.	p. 2 (summary only)
Selection process	8	Specify the methods used to decide whether a study met the inclusion criteria of the review, including how many reviewers screened each record and each report retrieved, whether they worked independently, and if applicable, details of automation tools used in the process.	p. 2
Data collection process	9	Specify the methods used to collect data from reports, including how many reviewers collected data from each report, whether they worked independently, any processes for obtaining or confirming data from study investigators, and if applicable, details of automation tools used in the process.	p. 3
Data items	10a	List and define all outcomes for which data were sought. Specify whether all results that were compatible with each outcome domain in each study were sought (e.g. for all measures, time points, analyses), and if not, the methods used to decide which results to collect.	p. 3
	10b	List and define all other variables for which data were sought (e.g. participant and intervention characteristics, funding sources). Describe any assumptions made about any missing or unclear information.	pp. 3–5; Table 1; Table S1
Study risk of bias assessment	11	Specify the methods used to assess risk of bias in the included studies, including details of the tool(s) used, how many reviewers assessed each study and whether they worked independently, and if applicable, details of automation tools used in the process.	p. 3
Effect measures	12	Specify for each outcome the effect measure(s) (e.g. risk ratio, mean difference) used in the synthesis or presentation of results.	p. 3
Synthesis methods	13a	Describe the processes used to decide which studies were eligible for each synthesis (e.g. tabulating the study intervention characteristics and comparing against the planned groups for each synthesis (item #5)).	pp. 2–5
	13b	Describe any methods required to prepare the data for presentation or synthesis, such as handling of missing summary statistics, or data conversions.	p. 3
	13c	Describe any methods used to tabulate or visually display results of individual studies and syntheses.	p. 3; Figures 3–7
	13d	Describe any methods used to synthesize results and provide a rationale for the choice(s). If meta-analysis was performed, describe the model(s), method(s) to identify the presence and extent of statistical heterogeneity, and software package(s) used.	p. 3
	13e	Describe any methods used to explore possible causes of heterogeneity among study results (e.g. subgroup analysis, meta-regression).	p. 3; Table 2; Supplementary Table S5
	13f	Describe any sensitivity analyses conducted to assess robustness of the synthesized results.	p. 3; Figure S1
Reporting bias assessment	14	Describe any methods used to assess risk of bias due to missing results in a synthesis (arising from reporting biases).	p. 3
Certainty assessment	15	Describe any methods used to assess certainty (or confidence) in the body of evidence for an outcome.	p. 3
**RESULTS**			
Study selection	16a	Describe the results of the search and selection process, from the number of records identified in the search to the number of studies included in the review, ideally using a flow diagram.	p. 4; Figure 1
	16b	Cite studies that might appear to meet the inclusion criteria, but which were excluded, and explain why they were excluded.	Supplementary Table S2

Section and Topic	Item #	Checklist item	Location where item is reported
Study characteristics	17	Cite each included study and present its characteristics.	pp. 4–5; Table 1; Table S1
Risk of bias in studies	18	Present assessments of risk of bias for each included study.	pp. 5–6; Figure 2
Results of individual studies	19	For all outcomes, present, for each study: (a) summary statistics for each group (where appropriate) and (b) an effect estimate and its precision (e.g. confidence/credible interval), ideally using structured tables or plots.	pp. 6–8; Figures 3–7
Results of syntheses	20a	For each synthesis, briefly summarise the characteristics and risk of bias among contributing studies.	pp. 4–6; Table 1; Figure 2
	20b	Present results of all statistical syntheses conducted. If meta-analysis was done, present for each the summary estimate and its precision (e.g. confidence/credible interval) and measures of statistical heterogeneity. If comparing groups, describe the direction of the effect.	pp. 6–9; Figures 3–7; Table 2
	20c	Present results of all investigations of possible causes of heterogeneity among study results.	pp. 8–9; Table 2; Supplementary Table S5
	20d	Present results of all sensitivity analyses conducted to assess the robustness of the synthesized results.	pp. 7–8; Figure S1
Reporting biases	21	Present assessments of risk of bias due to missing results (arising from reporting biases) for each synthesis assessed.	p. 9 (not assessed; <10 studies)
Certainty of evidence	22	Present assessments of certainty (or confidence) in the body of evidence for each outcome assessed.	p. 5; Supplementary Table S4
**DISCUSSION**			
Discussion	23a	Provide a general interpretation of the results in the context of other evidence.	pp. 9–10
	23b	Discuss any limitations of the evidence included in the review.	p. 10
	23c	Discuss any limitations of the review processes used.	p. 10
	23d	Discuss implications of the results for practice, policy, and future research.	pp. 9–10
**OTHER INFORMATION**			
Registration and protocol	24a	Provide registration information for the review, including register name and registration number, or state that the review was not registered.	p. 2 (PROSPERO CRD420261432477)
	24b	Indicate where the review protocol can be accessed, or state that a protocol was not prepared.	p. 2 (PROSPERO CRD420261432477)
	24c	Describe and explain any amendments to information provided at registration or in the protocol.	Not reported
Support	25	Describe sources of financial or non-financial support for the review, and the role of the funders or sponsors in the review.	p. 11
Competing interests	26	Declare any competing interests of review authors.	p. 11
Availability of data, code and other materials	27	Report which of the following are publicly available and where they can be found: template data collection forms; data extracted from included studies; data used for all analyses; analytic code; any other materials used in the review.	p. 11
